# Bilateral invasive lobular breast cancer in a female teenager: a rare finding of a common disease - case report and review of literature

**Published:** 2010-07-19

**Authors:** Enow-Orock George Enownchong, Egbe Obinchemti Thomas, Achidi Eric Akum, Asonganyi Etienne Defang, Ndom Paul, Fongang Emmanuel, Ndumbe Peter

**Affiliations:** 1 Department of Biological Sciences, University of Buea, Pathology Service, General Hospital, Yaoundé, Cameroon; 2 Department of Medicine,University of Buea, South-West Region, Cameroon; 3 District Hospital, Kumba, South-West region, Cameroon; 4 Medical Oncology service, General Hospital Yaoundé, Cameroon; 5 Radiology service, General Hospital Yaoundé, Cameroon

**Keywords:** Cancer, bilateral, lobular, carcinoma, teenager, rare

## Abstract

Abstract Management of cancer patients in low-resource communities presents enormous challenges. Breast cancer is a public health problem in Cameroon and occurs mostly in elderly women. The predominant histological type is a duct carcinoma. Lobular carcinoma in teenagers is rare. In this report we present a case of bilateral invasive lobular carcinoma of the breast that was confirmed on biopsies in a 22-year-old female. We present this rare finding and review the pathological, clinical and radiographic challenges of the disease. Nodules in the breast from patients of any age should be submitted for histology. Public education is beneficial and should be intensified.

## Patient and case report

IM is a 22-year-old seamstress who reported to a regional hospital following media sensitization on cancer with “thick patches” in both breasts noticed about a year earlier. Physical examination revealed a teenager of normal weight, with “bumpy” areas in both breasts. The “bumpy” areas were ill-defined, non-adherent and not tender “nodularities” in the upper-inner quadrant and the upper-outer quadrant of the right and left breasts respectively. No axillary nodes were palpated. No mammogram or echography was done. The rest of physical and investigative examinations were normal. A diagnosis of bilateral fibroadenoma was made.

Pathology examination showed two specimens of 4.0cm average diameter each with greyish, poorly-delimited, firm multiple foci on sections. Microscopy on Hematoxyline and Eosine-stained slides showed lobules filled with, and dilated by multiple layers of atypical small round cells with hyperchromatic, irregular nuclei and clear cytoplasm. The stroma was invaded in “Indian file”. Immuno-histochemistry was not done. A diagnosis of bilateral invasive moderately-differentiated (grade II) lobular carcinoma (ILC) was made and the patient referred.

## Discussion

In Cameroon, breast cancer is the commonest malignancy [1] that affects mainly females aged 35 to 54 years and predominantly a ductal type (70%). Invasive lobular carcinoma (ILC) in Cameroon (9.5 %) affects mainly women above 55 years and is rare in teenagers [2]. A bilateral disease in this age group as we found in our case is even rarer.

It is usually difficult to diagnose ILC early due to lack of typical radiographic and clinical signs. The vague findings may be confused with benign breast changes, especially in the younger patient. Our case exemplifies the challenges of health-care in general and cancer in particular in low-resource communities. Public sensitization is important as most patients go to hospital only when in pain. It is also important that all surgical specimens, even from patients with no known risks for cancer be sent for histology. Though age is a significant risk for cancer, younger persons are increasingly becoming affected by cancers traditionally known to be peculiar to the elderly [2].

In their original article on lobular carcinoma, Foote and Stewart [3] in 1941 established the term “invasive lobular carcinoma”. The histologic features described were an associated desmoplastic stromal reaction and linear arrangement of cells— Indian file. Invasive lobular carcinoma (ILC) of the breast is the second most common type of primary breast cancer, accounting for 10-15% of cases [4].

In a stage-matched comparison of outcomes between ILC and invasive duct carcinoma (IDC) by El Saghir et al [5], the median age at presentation was 66 years for ILC and 60 years for IDC (p <0.001). Christopher et al report that the proportion of breast cancers with a lobular component increased from 9.5 percent in 1987 to 15.6 percent in 1999 [6].

Risk factors for breast cancer have been established in previous studies [7]. Paul et al concluded that lobular carcinoma in-situ (LCIS) is associated with increased risk of subsequent invasive disease to about 7.1% at 10 years [8]. Although lobular structures are infrequent in the normal male, sporadic cases of ILC have been described in male breast [9].

In comparison with IDC, ILC is significantly more likely to occur in older patients, larger, bilateral, multifocal and multicentric [10]. Biologically ILC is more often PR+, ER+, diploid and HER-2, p53, and epidermal growth factor receptor (EPGFR) negative compared to IDC [11]. In some cases like ours, no risk factors are identified and physical exam may be non-specific.

Ultrasound and MRI have been shown to be more sensitive than mammography for invasive cancer, but with the risk of overestimating the extent of the tumour. Combined mammography, clinical exam, and MRI are more sensitive at detecting ILC than any other individual test or combination usually a speculated mass with asymmetric density and no definable margins nor mass [12].

Contrast-enhanced MRI is highly sensitive in detecting ILC and its extent, which is of value in pre-operative planning as well as the diagnosis of multifocal or contra lateral involvement [13]. The Computer Aided Detection (CAD) system has increased the likelihood of detecting breast cancer, to about 91% [14, 15]. Because of non-specific findings, infrequency of micro-calcification, and slow growth, ILC is often detected only in late stages [16] though Arpino [11] found the five-year disease-specific survival to be significantly better for patients with ILC compared to IDC. ILC commonly metastasizes to bone, gastrointestinal tract and ovary and studies have shown that ILC is the most common extra-genital neoplasm that metastasizes to gynaecologic organs [16].

To determine the value of cytology in the differential diagnosis of ductal and lobular carcinoma of the breast, de las Morenas et al [17] showed that amongst various parameters, chromatin pattern (P<.0001), nuclear size (P<.004) and cell size (P <.004) showed statistically significant differences between the two groups. Based on this study, the presence of coarsely granular chromatin, nuclear size >44 microm2 and cell size > 82 microm2 are the only features diagnostic of ductal versus lobular carcinoma [17]. ILC is more likely to be >2 cm in size (43.1% vs. 32.6%, p <0.001), lymph node positive (36.8% vs. 34.4%, p <0.001) and ER+ (93.1% vs. 75.6%, p <0.001) and may contain signet ring cells [18]. Majority are histologic grade 2 (as in this case). Fine needle aspiration cytology often does not yield much information in lobular cancer due to the sparseness of malignant cells, and wide-bore needle biopsy is more recommended.

There is a bias toward treating ILC with aggressive therapy with some series indicating a 3-6 fold excess in patients receiving mastectomy compared with breast conservation therapy (BCT). In a comparative study between BCT and mastectomy for ILC, Singletary et al [19] found that less invasive treatment options are becoming widely used for the disease with outcomes similar to more aggressive treatment. Due to the infiltrative growth pattern and frequent discontinuities, there is a higher incidence of resection margin involvement in ILC with more intra-surgical conversion to mastectomy [19].

Management of cancer patients all over the world is fraught with numerous challenges. These challenges are more acute in low resource communities like ours. This case highlights the importance of public education and multidisciplinary approach in cancer care, through referrals. It also brings to light the difficulty that health professionals face in the absence of basic facilities like ultrasound, mammography and immunohistochemistry in this case.

## Conclusion

Cancer is a public health problem in Cameroon, and increasingly affecting younger persons. Breast cancer is the commonest cancer in Cameroon and the predominant histologic type is a ductal carcinoma. ILC is mainly a disease of elderly females, though no age is immune, even in the absence of any risk factors, as in this teenage female with bilateral disease. Management of cancer patients in low-resource communities faces many challenges; therefore public and health personnel education should be intensified. All breast nodules from patients of any age should be submitted for histology. Developing countries need to invest more resources in the health sector.

## Authors’ contributions

EOGE: pathologist who analysed the biopsy specimens and reviewed the pathology literature **NPaul**: medical oncologist to whom patient was referred and reviewed the medical oncology literature. **AED**: referred the patient after biopsy to the pathologist. **AEA**: chemical pathologist who

## Competing interests

The authors declare no competing interests
